# What really happens in the home: a comparison of parent-reported and observed tooth brushing behaviors for young children

**DOI:** 10.1186/s12903-019-0725-5

**Published:** 2019-02-21

**Authors:** Molly Martin, Genesis Rosales, Anna Sandoval, Helen Lee, Oksana Pugach, David Avenetti, Gizelle Alvarez, Anabelen Diaz

**Affiliations:** University of Illinois at Chicago, 1747 West Roosevelt Road, Room 547, M/C 275, Chicago, IL 60608 USA

**Keywords:** Dental care for children, Healthcare disparities, Oral health, Child health, Prevention, Toothbrushing

## Abstract

**Background:**

Most studies of tooth brushing behaviors rely on self-report or demonstrations of behaviors conducted in clinical settings. This study aimed to determine the feasibility of objective assessment of tooth brushing behaviors in the homes of high-risk children under three years old. We compared parent self-report to observations to determine the accuracy of self-report in this population.

**Methods:**

Forty-five families were recruited from dental and medical clinics and a community social service agency. Research staff asked questions about oral health behaviors and observed tooth brushing in the homes. Brushing was also video-recorded. Video-recordings were coded for brushing behaviors by staff that did not collect the primary data; these abstracted data were compared to those directly observed in homes.

**Results:**

Most families were Hispanic (76%) or Black (16%) race/ethnicity. The majority of parents had a high school education (42%) or less (24%). The mean age of children was 21 months. About half of parents reported brushing their child’s teeth twice a day (58%). All parents tried to have their children brush, but three children refused. For brushing duration, 70% of parents reported differently than was observed. The average duration of brushing was 62.4 s. Parent report of fluoride in toothpaste frequently did not match observations; 39% said they used toothpaste with fluoride while 71% actually did. Sixty-eight percent of parents reported using a smear of toothpaste, while 61% actually did. Brushing occurred in a variety of locations and routines varied. Abstracted data from videos were high in agreement for some behaviors (rinse with water, floss used, brushing location, and parent involvement: Kappa 0.74–1.0). Behaviors related to type of brushing equipment (brushes and toothpaste), equipment storage, and bathroom organization and clutter had poor to no agreement.

**Conclusions:**

Observation and video-recording of brushing routines and equipment are feasible and acceptable to families. Observed behaviors are more accurate than self-report for most components of brushing and serve to highlight some of the knowledge issues facing parents, such as the role of fluoride.

## Background

From 2015 to 2016, the prevalence of total dental caries in United States youth aged 2–19 years was 43.1%; almost 18% of these began before the age of six [1]. The burden of caries is not distributed evenly. Low-income and minority populations experience disproportionately higher caries prevalence and morbidity rates [1–3]. These disparities are frequently attributed to inadequate dental coverage and utilization, insufficient exposure to fluoride, unhealthy dietary choices, and poor oral hygiene [4–6]. Preventive interventions that target these factors in very young children can potentially reduce future pain, infections, malnutrition, speech difficulties, poor school performance, cosmetic problems, and quality of life that are associated with caries [7–9]. While some of these factors are measured using objective data sources such as insurance and billing records, most rely on self-report or demonstrations of behavior conducted in clinical settings. But what do we actually know about what happens in the home regarding oral hygiene in these high-risk populations for very young children?

COordinated Oral health Promotion (CO-OP) Chicago is part of a health disparities research collaborative funded by the National Institutes of Health. In preparation for a trial testing a community-based behavioral oral health intervention with young children, CO-OP Chicago conducted several planning studies. These included a survey of parents in pediatric dental clinic waiting rooms and a pilot study to test recruitment and data collection protocols. Some of these participants received home observations where brushing behaviors were objectively assessed; those data are the focus of this analysis. Because we could find no published reports of objective assessment of brushing behaviors in the homes of children under the age of three, we first tested the feasibility of observing brushing behaviors in the homes. We then compared parent self-report to observations of tooth brushing to determine the accuracy of self-report in this population. Finally, observations in the homes allowed us to describe the environments where brushing occurs and the equipment and products used. These data help us to better understand the situations and environments families navigate and provide strategies for how to assess tooth brushing behaviors in high-risk populations.

## Methods

Participants were recruited from four sites: a university pediatric dental clinic in a large medical district and a community pediatric dental clinic (referred to as pediatric dental clinic families), and a pediatric medical clinic in a large medical district and a Special Supplemental Nutrition Program for Women, Infants, and Children (WIC) center run by the Chicago Department of Public Health (referred to as medical clinic and WIC families). Research assistants (RAs) approached parents with young children in the site waiting rooms and described the study. The RAs were female, bilingual in English and Spanish, and of Hispanic ethnicity. To qualify, the following inclusion criteria had to be met: 1) Parent at least 18 years old, 2) Child under the age of three (self-report), 3) Child had to have at least one tooth, 4) Parent had to live with child at least five days out of the week, and 5) Parent had to speak English or Spanish.

Out of the 479 parents approached from October 6, 2016, to May 26, 2017, 190 met inclusion criteria, 76 agreed to a home observation, and 45 completed a home observation. The sample size of 45 was considered to be sufficient to answer the primary question of feasibility. A pair of RAs conducted the home observations. RAs practiced data collection with volunteers until supervisors determined their adherence to the data collection protocol was sufficient. After obtaining informed consent, parents were asked questions about tooth brushing frequency and duration, toothpaste use, caregiver self-efficacy, caregiver support, dental access, and medical/dental insurance. Parents were then asked to demonstrate how they brush their child’s teeth. Tooth brushing duration was recorded as the moment the toothbrush entered the child’s mouth until the caregiver stated the tooth brushing was completed. Equipment (type of toothpaste and toothbrush, quality of toothbrush, mouthwash, cup), the physical space, and the brushing process were visually observed and documented by RAs. The brushing process was also video recorded. One RA entered data on a computer while the other timed and recorded behaviors. Finally, RAs asked questions to document family demographics. Participants were compensated with a cash incentive ($25 or $40 depending which phase they were a part of) and were given toothbrushes and an oral health information sheet at the end of the visit. Data was collected using Qualtrics (Qualtrics; Copyright 2017; Provo, Utah; Oct. 6, 2016-May. 26, 2017) and REDCap electronic data capture tools [10].

This study was approved by the University of Illinois at Chicago Institutional Review Board (Protocols 2015–0815 and 2016–0773) and the Chicago Department of Public Health Institutional Review Board (#16–06). Adult participants provided written informed consent and parental permission; child participants were too young to provide assent.

Descriptive statistics (mean/frequencies) were used to summarize variables. Some of the more detailed response categories (e.g., education level) were collapsed for reporting purposes and based on available responses. Oral health behaviors that were assessed through both caregiver self-report and by RA observation were coded as concordant (self-report matched observation) or not, and then compared to demographics and other oral health behaviors using Fishers Exact Test to identify statistically significant associations. Video recordings of brushing routines were viewed by research staff who were not present during the home observations; these staff independently coded video observations in a separate database. Cohen’s kappa statistic was used to measure the agreement between video-captured data from the home visit and data recorded by RAs in the home. Analyses were conducted in SAS 9.4 software (SAS Proprietary Software 9.4, Copyright© 2016. Cary, NC: SAS Institute Inc). Significance of statistical results were determined with critical values below 0.05.

## Results

Participants reflected the demographics of the sites they were recruited from (Table 1). The majority reported Hispanic ethnicity (76%), with most of the rest identifying as Black (16%). Over half of parents had a high school education (42%) or less (24%). The mean age of children was 21 months (SD = 6, range 9–36 months). Recent changes to Medicaid managed care confused many parents about their type of insurance, but many knew they had public medical insurance and the recruitment sites serve mainly patients on public insurance. Many parents did not have their own medical insurance (49%), and the majority of parent health policies did not cover dental (52%).

**Table 1:** Study Participant Demographics

	Total Sample *N* = 45	Pediatric Dental Clinic Families *N* = 25	Medical Clinic and WIC Families *N* = 20
Parent female (%)	43 (95.6)	25 (100.0)	18 (90.0)
Parent age in years, mean (SD)	31.2 (6.1)	32.8 (4.6)	29.3 (7.2)
Child female (%)	31 (68.9)	18 (72.0)	13 (65.0)
Child age in months, mean (SD)	21.1 (6.3)	21.5 (6.1)	20.7 (6.8)
Parent race (%)			
White	5 (11.1)	3 (12.0)	2 (10.0)
Black	7 (15.5)	1 (4.0)	6 (30.0)
Other	33 (73.3)	21 (84.0)	12 (60.0)
Parent Hispanic (%)	34 (75.5)	21 (84.0)	13 (65.0)
*Mexican*	*27 (79.4)*	*17 (81.0)*	*10 (76.9)*
*Other Hispanic*	*6 (17.6)*	*4 (19.0)*	*2 (15.4)*
Parent education (%)			
Less than high school	11 (24.4)	7 (28.0)	4 (20.0)
High school/GED	19 (42.2)	11 (44.0)	8 (40.0)
Some college	6 (13.3)	1 (4.0)	5 (25.0)
College degree or higher	9 (20.0)	6 (24.0)	3 (15.0)
Child medical insurance type (%)			
Public	26 (57.8)	13 (52.0)	13 (65.0)
Private	1 (2.2)	1 (4.0)	0 (0.0)
Not sure public or private*	18 (40.0)	11 (44.0)	7 (35.0)
Does child’s medical insurance cover dental? (%)			
Yes	40 (88.9)	23 (92.0)	17 (85.0)
No	3 (6.6)	2 (8.0)	1 (5.0)
Don’t know	2 (4.4)	0 (0.0)	2 (10.0)
Parent medical insurance (%)			
Public	14 (31.1)	7 (28.0)	7 (35.0)
Private	4 (8.9)	2 (8.0)	2 (10.0)
Not sure public or private	5 (11.1)	1 (4.0)	4 (20.0)
No insurance	22 (48.8)	15 (60.0)	7 (35.0)
Does parent health insurance cover dental? (%)**			
Yes	17 (40.5)	6 (27.3)	11 (55.0)
No	22 (52.4)	14 (63.6)	8 (40.0)
Don’t know	3 (7.1)	2 (9.1)	1 (5.0)

*Recent changes to Medicaid managed care confused many parents about the type of insurance. The sites these families were recruited from serve mainly families on Medicaid

**N = 42 in Total Sample; *N* = 22 in Clinic Families

About half of parents reported brushing their child’s teeth twice a day (58%), although brushing frequency was higher in parents recruited from dental clinics (Table 2). Some parents (38%) claimed daily activities got in the way of brushing and 27% of parents had very little or no help with their child’s oral care. Many of the children had not been to the dentist yet (27%), and several children already had experienced caries (7%) per parent self-report. Almost all parents brushed their own teeth at least twice a day, but 38% had not been to the dentist themselves in the past year, and 51% reported the overall condition of their mouth and teeth was fair/poor.

**Table 2:** Study Participant Oral Health Characteristics

	Total Sample *N* = 45 (%)	Pediatric Dental Clinic Families *N* = 25 (%)	Medical Clinic and WIC Families *N* = 20 (%)
Child brushing frequency			
Never	2 (4.4)	0 (0.0)	2 (10.0)
Sometimes but not every day	1 (2.2)	0 (0.0)	1 (5.0)
Once a day	10 (22.2)	4 (16.0)	6 (30.0)
Twice a day	26 (57.8)	17 (68.0)	9 (45.0)
More than twice a day	6 (13.3)	4 (16.0)	2 (10.0)
How often do activities of daily life get in way of caring for child’s teeth?			
All/Most of the time	8 (17.8)	6 (24.0)	2 (10.0)
Some of the time	9 (20.0)	2 (8.0)	7 (35.0)
Rarely/Never	28 (62.2)	17 (68.0)	11 (55.0)
How often does your family help you care for child’s teeth?			
All/Most of the time	21 (46.7)	9 (36.0)	12 (60.0)
Some of the time	12 (26.7)	9 (36.0)	3 (15.0)
Rarely/Never	12 (26.7)	7 (28.0)	5 (25.0)
When did child last go to dentist?			
6 months or less	31 (68.9)	20 (80.0)	11 (55.0)
6 months -- 1 year ago	1 (2.2)	1 (4.0)	0 (0.0)
1 year -- 2 years ago	1 (2.2)	0 (0.0)	1 (5.0)
Never has been	12 (26.7)	4 (16.0)	8 (40.0)
Child has had a cavity or tooth decay	3 (6.7)	3 (12.0)	0 (0.0)
What kind of water does child drink?			
Tap water	1 (2.2)	0 (0.0)	1 (5.0)
Filtered water from tap	11 (24.4)	5 (20.0)	6 (30.0)
Bottled water	28 (62.2)	16 (64.0)	12 (60.0)
Other	5 (11.1)	4 (16.0)	1 (5.0)
Parent brushing frequency			
Never	0 (0.0)	0 (0.0)	0 (0.0)
Sometimes but not every day	1 (2.2)	0 (0.0)	1 (5.0)
Once a day	0 (0.0)	0 (0.0)	0 (0.0)
Twice a day	29 (64.4)	17 (68.0)	12 (60.0)
More than twice a day	15 (33.3)	8 (32.0)	7 (35.0)
When did parent last go to dentist?			
6 months or less	17 (37.8)	11 (44.0)	6 (30.0)
6 months -- 1 year ago	11 (24.4)	6 (24.0)	5 (25.0)
1 year -- 2 years ago	10 (22.2)	5 (20.0)	5 (25.0)
More than 2 years ago	6 (13.3)	3 (12.0)	3 (15.0)
Never has been	1 (2.2)	0 (0.0)	1 (5.0)
Condition of parent’s mouth and teeth			
Very good	3 (6.7)	2 (8.0)	1 (5.0)
Good	19 (42.2)	8 (32.0)	11 (55.0)
Fair	18 (40.0)	11 (44.0)	7 (35.0)
Poor	5 (11.1)	4 (16.0)	1 (5.0)

During the home observations, all parents tried to have their children brush, but three children refused to let the brush touch their mouth. As shown in Table 3, all parents reported they helped their child brush, and they all did. For brushing duration overall, 70% of parents reported differently than was observed. Sixteen percent of parents reported brushing 30 s or less, and 14% actually did. Parents were also accurately reporting 1–2 min of brushing. Parents were less accurate in the other categories: 22% said they brushed more than two minutes, but only 2% actually did. The average duration of brushing was 62.4 s (SD = 34.2, range 0–138). Parent reports of brushing time compared to observations were less likely to agree for parents that selected “other” as race (*p* < 0.01), were Hispanic (*p* < 0.01), or had a high school education (*p* = 0.02).

**Table 3:** Self-Reported and Observed Child Brushing Behaviors

	Parent REPORTED *N* = 45 (%)	OBSERVED *N* = 45 (%)	OBSERVED	
			Pediatric Dental Clinic Families *N* = 25 (%)	Medical Clinic and WIC Families *N* = 20 (%)
Parent or adult helps child brush teeth^1^		44 (100.0)	25 (100.0)	19 (100.0)
Yes, sometimes	7 (16.3)			
Yes, most of the time	5 (11.6)			
Yes, always	31 (72.0)			
How long are child’s teeth brushed for^2^				
Does/did not brush	2 (4.4)	3 (7.0)	0 (0.0)	3 (15.0)
30 s or less	7 (15.6)	6 (14.0)	3 (13.0)	3 (15.0)
> 30 s to 1 min	8 (17.8)	15 (34.9)	7 (30.4)	8 (40.0)
> 1 min to 2 min	18 (40.0)	18 (41.9)	12 (52.2)	6 (30.0)
> 2 min	10 (22.2)	1 (2.3)	1 (4.3)	0 (0.0)
Child uses toothpaste	41 (91.1)	41 (91.1)	24 (96.0)	17 (85.0)
Does toothpaste have fluoride? ^3^				
Yes	16 (39.0)	30 (71.4)	21 (84.0)	9 (52.9)
No	10 (24.4)	12 (28.6)	4 (16.0)	8 (47.1)
Don’t know	15 (36.6)			
How much toothpaste does child use? ^4^				
Full load	0 (0.0)	1 (2.4)	0 (0.0)	1 (5.9)
Half load	2 (4.9)	2 (4.8)	2 (8.3)	0 (0.0)
Pea	11 (26.8)	8 (19.5)	4 (16.7)	4 (23.5)
Smear	28 (68.3)	25 (61.0)	15 (62.5)	10 (58.8)

1: *N* = 43 in Parent Reported; *N* = 44 in Observed; *N* = 19 in Medical Clinic Observed

2: *N* = 43 in Observed; *N* = 23 in Dental Clinic Observed

3: *N* = 41 in Parent Reported; *N* = 42 in Observed; *N* = 17 in Medical Clinic Observed. Note that one parent reported the child did not use toothpaste but then used toothpaste when brushing

4: *N* = 41 in Parent Reported; *N* = 41 in Observed; *N* = 24 in Dental Clinic Observed; *N* = 17 in Medical Clinic Observed. Note that one parent reported the child did not use toothpaste but then used toothpaste when brushing. Another child began brushing before the quantity of toothpaste could be observed

Two parents reported that their children used toothpaste, but no toothpaste was available during the visit. Parent report of fluoride in toothpaste did not match observations for 39%. Thirty-nine percent of parents said their child’s toothpaste had fluoride, and another 37% did not know. However, 71% of the toothpaste observed had fluoride. Parents who reported that the activities of daily living got in the way of brushing some or all of the time were less likely to be concordant between self-report and observations of toothpaste fluoride (*p* < 0.01). Participants recruited from the dental clinics were more likely to have fluoridated toothpaste than the other participants (84% compared to 53%). Parent report differed from observations of the quantity of toothpaste for 33% of families. The majority of parents (68%) reported, and did (61%), use a smear of toothpaste which is the appropriate amount for children under the age of three.

Table 4 shows additional details observed in the homes. Most parents brushed in the bathroom (82%), but other locations included kitchens, family rooms, and bedrooms. The majority of the time (61%), children were standing, frequently on the closed toilet. Some parents had children spit (50%) and rinse (63%). No children were sharing toothbrushes and most had child-sized toothbrushes (96%) that were in good condition. Overall, the brushing areas were clean with only mild clutter. RAs attempted to document what portions of children’s teeth were brushed and were successful in 80% of cases.

**Table 4:** Characteristics of Observed Brushing Areas and Behaviors

	OBSERVATIONS *N* = 45 (%)
*Brushing Behaviors*	
Brushing occurred in bathroom	37 (82.2)
Brushing occurred with child standing^1^	27 (61.4)
Child spit after brushing^2^	20 (50.0)
Child rinsed after brushing with water^2^	25 (62.5)
Child rinsed with mouthwash^1^	1 (2.2)
Child used floss	2 (4.4)
How much of teeth brushed?	
None	6 (13.3)
Anterior	33 (75.0)
Posterior	17 (37.8)
Outside portions	19 (42.2)
Inside portions	15 (33.3)
Could not properly see to assess	5 (11.1)
*Brushing Equipment*	
Rinsing cup present	14 (31.1)
Child has own toothbrush (not shared)	45 (100.0)
Type of child toothbrush	
Child sized	43 (95.6)
Adult	1 (2.2)
Electronic	1 (2.2)
Toothbrush condition	
Looks new	27 (60.0)
Bristles in good shape	10 (22.2)
Bristles bending/starting to wear	7 (15.6)
Unable to assess – brush capped	1 (2.2)
Type of toothpaste^1^	
None	2 (4.5)
Infant/Child without fluoride	12 (27.3)
Infant/Child with fluoride	24 (54.6)
Adult with fluoride	6 (13.6)
*Brushing Environment*	
Number of toothbrushes visible, mean (SD)^2^	3.3 (2.9)
Where toothbrushes stored	
In tooth brush holder	12 (26.7)
On sink or counter	15 (33.3)
Laying in cabinet or on shelf	9 (20.0)
Bedroom	1 (2.2)
Could not tell	8 (17.8)
Number of tubes of toothpaste visible, mean (SD)^3^	1.5 (1.2)
Level of clutter at sink	
No clutter	17 (37.8)
Some clutter	22 (48.9)
Not observable because not in bathroom	6 (13.3)
Level of cleanliness of tooth brushing area	
Very clean (spotless)	26 (57.8)
Moderately clean	9 (20.0)
Dirty	5 (11.1)
Not observable because not in bathroom	5 (11.1)

1: *N* = 44. 2: *N* = 40. 3: *N* = 41

In total, 36 videos (80%) were coded and compared to home observations of brushing because one video was in Polish and eight others did not consent to video recording. Certain behaviors (rinse with water, floss used, brushing location, and parent involvement) were very high in agreement (Kappa 0.74–1.0). Other behaviors (child brushing position, brushing time, spit after, and rinsing cup present) had a moderate agreement (Kappa between 0.50–0.63). Poor to no agreement (Kappa < 0.23) was noted for type of toothbrush, toothbrush condition, if child had own toothbrush, where toothbrushes were stored, use of toothpaste, type of toothpaste, use of mouthwash, level of sink clutter, and cleanliness of sink.

## Discussion

This small sample of urban low-income families provides a unique glimpse into what really happens in the homes of young children under the age of three. Most studies to date in this age group rely on self-report or observations conducted in a clinical or research setting. While these settings attempt to replicate the home environment, they are artificial and limited in their comparability to real homes. Homes are comforting and familiar to young children, making them more likely to demonstrate their routines accurately [11]. Observations in homes also accommodate the tremendous variability in home layouts and routines, allowing for the recognition of physical barriers (e.g., small bathrooms, limited counter space) and objective verification of equipment and supplies. Our study demonstrates that observation of brushing routines and equipment is acceptable to some families and feasible from a data collection standpoint. Even video-recording of behaviors was accepted although this proved to be logistically challenging in many of the small bathrooms. The data collected from objective observations provided additional detail and allowed for verification of parent-reported accuracy.

While not objectively verified, our sample’s parent report of brushing frequency was comparable to results from Washington State and Australia [12, 13]. In National Health and Nutrition Examination Survey (NHANES) 2014 data, 62% of parents/caregivers of children 3–4 years old report brushing twice a day or more which is comparable to our results even though our age range is under three years old [14]. Commonly reported barriers to brushing are lack of time and an uncooperative child [15], emphasizing the critical role of the caregiver in the brushing process [16]. Parent assistance with brushing was reported as a universal practice for participants in our study, although some parents reported they did not always help. The results of other studies with young children suggest these children are likely expected to brush on their own frequently. Parents in a rural Washington State community sample reported 10% of children under the age of five brushed without assistance [13]. In a small sample of two-year-old children in Scotland, home video-recording of brushing showed that the majority of brushing was done by children alone [17]. We do not know if this was because they thought the children were competent to brush on their own or because the parents did not have the time or interest.

Our sample’s average brushing time was 62.4 s, which is similar to higher income mainly non-Hispanic white children observed in a dental clinic in Seattle where the average brushing duration observed was 71 s [16]. The challenge with these data is brushing duration is not always a continuous activity. Children start and stop, often removing and re-inserting the brush multiple times [17]. This is not necessarily a bad thing; although the toothbrush may be removed from the mouth, fluoride toothpaste has the opportunity to remain on the teeth during these pauses. Parent report of brushing duration varied in its accuracy. Very likely parents misjudge total time, but they also may vary in their definitions of the start and end points of brushing. Our data suggest that objective measurement of brushing is optimal, and that clear start and stop points for brushing should be defined from the start.

While fluoridated toothpaste is recommended for young children [18], its use is rarely measured [14, 19]. Sixty-one percent of our parents reported they did not use fluoridated toothpaste or did not know if they did for their children, and yet 74% of the child toothpaste observed had fluoride. We expected a smaller proportion of families would use fluoridated toothpaste because of the robust marketing of fluoride-free toothpaste to babies. We could find no other literature demonstrating this lack of concordance between reported and observed use of fluoride toothpaste. Our results suggest that in this low-income population, many parents were unaware of fluoride recommendations, controversies, and advertising and therefore were unintentional in their use of fluoridated toothpaste. Parents accurately reported the quantity of toothpaste used and mostly this was in alignment with recommendations for the child’s age [20, 21]. This is in contrast to research conducted by others, where parents incorrectly reported the amount of toothpaste used and consistently applied larger quantities of toothpaste than was recommended [22, 23]. We expected more chaotic homes and more sharing of equipment than was observed. This is likely because the families that volunteered for the study were motivated regarding oral health and knew we were coming to observe these behaviors.

We saw interesting differences between families recruited from pediatric dental clinics and medical clinics. We assumed families from pediatric dental clinics would be more aware of oral health recommendations, although they might have been in the dental clinics because their children already had oral health problems. Our numbers are small but suggest slightly worse caries and less support for families from the dental clinics.

While the majority of families allowed video recording, the collection of adequate video data for abstraction was challenging due to the small bathrooms; therefore, we ultimately decided video recordings were not necessary to document brushing behaviors and equipment for the CO-OP Chicago clinical trial. However, we recognize the advantage of video recordings to capture behavioral interactions, specifically parent-child behaviors. Our direct observations did not capture child-parent interactions during brushing, but this domain is very important in order to ensure proper brushing technique and behavior maintenance [15–17], suggesting a role for video recording in other studies.

We recognize this study has limitations. Families that allow us into their homes are assumed to be more motivated by oral health behaviors than general populations, especially families recruited from pediatric dental clinics, which is observed in our data. Our sample was not homogenous; families recruited from pediatric dental clinics reported more children had caries and that daily life interfered more with their ability to care for their child’s teeth compared to other parents. Families cleaned and prepared for the home observations, demonstrating the influence of social desirability. Social desirability can affect both self-report and behaviors. Also, as with most observed research, the responses of families that volunteer are often influenced by the process of being observed and may demonstrate volunteer bias. Because of this, we assume behaviors and self-reported rates of behaviors are actually worse than what we measured for average low-income families in Chicago. Another concern is that behaviors conducted under observation may be different from those that normally occur, although observations in natural settings such as the home have been shown to not be affected by the presence of an observer [11]. Our sample was primarily Hispanic, urban, and low-income which is not generalizable to other populations. Finally, our sample size was small.

Despite these limitations, our results demonstrate the feasibility of observing tooth brushing behaviors of young children in homes of low-income families. Observed behaviors are more accurate than self-report for most components of brushing and serve to highlight some of the knowledge issues facing parents, such as the role of fluoride. Home observation also opens a window into some of the creative ways low-income families carry out recommendations, such as how parents position children in the bathrooms or brush in other rooms. This must be placed into the context of community acceptability of home visits. Only 24% of eligible participants had an actual home observation conducted. Formative work conducted by CO-OP Chicago and others suggest a range of reasons why individuals agree or do not agree to home visits. Families appreciate the convenience and intimacy of home visits, but they are also afraid of being judged and are nervous (for safety reasons) to let strangers into their homes [24, 25]. Early tooth brushing with attention to family dynamics, proper technique and fluoride toothpaste protects against caries and establishes lifelong behaviors [26, 27]. Further research is needed to describe and support parenting behaviors regarding effective brushing in the home environment where these behaviors begin and are sustained.

## Abbreviations

CO-OP: COordinated Oral health Promotion
NHANES: National Health and Nutrition Examination Survey
RA: Research Assistant
WIC: Women Infant and Children
